# ΔNp53 and aging

**DOI:** 10.18632/aging.100609

**Published:** 2013-10-20

**Authors:** Shih-Chieh Lin, Dylan J. Taatjes

**Affiliations:** University of Colorado, Boulder, CO, 80303, USA

The initial discovery that p53 could cause accelerated aging in mice was made with an artificial truncated form of p53 [1]. The naturally occurring ΔNp53 isoform was studied in detail by the Scrable group [2]. Both studies had similar conclusions: when expressed together with WTp53, the ΔNp53 isoform caused accelerated aging. As a transcription factor, p53 binds DNA as a tetramer, and mixed expression of WTp53 and ΔNp53 was key to the accelerated aging phenotype. The requirement for mixed tetramers containing ΔNp53 and WTp53 complicates analysis of the ΔNp53 isoform, as co-expression of ΔNp53 and WTp53 will result in heterogeneity, including formation of WTp53 tetramers. The presence of WTp53 tetramers together with ΔNp53 tetramers and mixed ΔNp53:WTp53 tetramers precludes a reliable comparison of ΔNp53:WTp53 with WTp53. To circumvent this problem, we implemented a strategy, based upon the WTp53 tetramer structure [3], that ensured a 2:2 stoichiometry (ΔNp53:WTp53) while maintaining tetramer architecture. Our approach allowed a clear delineation of cellular changes specifically caused by ΔNp53:WTp53, compared with WTp53 [4]. In this editorial, we briefly summarize our results with ΔNp53:WTp53 and highlight some observations not discussed in the original manuscript, based in part upon interesting and relevant work published more recently. As expected, ΔNp53:WTp53 tetramers were stable and transcriptionally active, as revealed by recombinant expression and biochemical purification, in vitro transcription, and cell-based assays. ΔNp53:WTp53 tetramers lack only 39 amino acids in two proteins in the tetramer, and affects on cell cycle, growth, apoptosis, and senescence were largely identical with WTp53 (at least with the duration of our experiments). Gene expression profiles for ΔNp53:WTp53 vs. WTp53 were also very similar; however, many of the ~80 genes that showed 2-fold or greater expression changes (ΔNp53:WTp53 vs. WTp53) are implicated in aging (4). These data demonstrate that ΔNp53:WTp53 should not be considered “hyperactive” relative to WTp53; rather, ΔNp53:WTp53 is “neomorphic” in that it adopts new functionality distinct from WTp53. Our results with ΔNp53:WTp53 were largely consistent with mouse models, most notably with respect to p21/*CDKN1A* gene expression and the insulin signaling pathway. In addition to gene expression analyses, we completed a series of metabolomics experiments to help identify how WTp53 and ΔNp53:WTp53 differentially affect cell physiology. These data sets represent the most comprehensive analysis thus far completed for the human ΔNp53:WTp53 isoform. Collectively, the microarray and metabolomics data yielded clear predictions (validated by pathway analyses) about the cellular pathways disrupted by ΔNp53:WTp53 specifically. Among the most striking were links to mitochondrial function and the mTOR pathway, which influence longevity in organisms as diverse as yeast and mammals [5. 6]. Numerous intriguing questions emerge from the ΔNp53:WTp53 data [4]. Mitochondrial membrane potential and ATP levels were elevated in response to ΔNp53:WTp53 expression, relative to WTp53. How could this contribute to aging? Mitochondria with reduced membrane potential are preferentially degraded in a process known as mitophagy (mitochondrial autophagy). Persistent elevated membrane potential in cells expressing ΔNp53:WTp53 could interfere with mitophagy and normal mitochondrial maintenance and turnover. We hypothesize that this could accelerate a decline in overall mitochondrial function. Moreover, whereas WTp53 typically acts to enhance autophagy, ΔNp53:WTp53 expression likely inhibits autophagy, because the mTOR pathway (a well-established inhibitor of autophagy and mitophagy) remains active upon ΔNp53:WTp53 expression, in contrast to WTp53 [4].

Another interesting question involves potential links to mitochondrial unfolded protein response (UPR^mt^). The Auwerx group recently established that “mitonuclear protein imbalance” (i.e. altered balance between nuclear-encoded and mitochondria-encoded OXPHOS subunits) could induce UPR^mt^ in metazoans; notably, this response appears to promote longevity [7]. Houtkooper et al. demonstrated that mTOR pathway inhibition or reduced mitochondrial translation were potent inducers of UPR^mt^ (longevity-promoting). Some of our findings with ΔNp53:WTp53 vs. WTp53 converge on these same processes, but in an opposite manner. For example, the mTOR pathway is activated by ΔNp53:WTp53 expression. Also, the Auwerx group linked reduced expression of mitochondrial translation factors (e.g. Mrps5) specifically, and reduced mitochondrial translation generally, to UPR^mt^ [7]. By contrast, it is likely that mitochondrial translation is increased by ΔNp53:WTp53, based upon its ability to elevate MARS2 expression (the mitochondrial tRNA^met^ synthetase) about 2.5-fold relative to WTp53 (4). The longevity-promoting effects of UPR^mt^ were also linked to mitochondrial proteostasis (via expression of HSP-70 and HSP-60) and mitochondrial biogenesis [7]; however, these changes were not induced by ΔNp53:WTp53 [4]. The fact that ΔNp53:WTp53 retains many basic functions of WTp53 (e.g. growth arrest and apoptosis) while losing other functions (e.g. mTOR inhibition) sets up a series of cellular changes that are only beginning to be understood in detail (see Figure). Furthermore, aging is a complex process involving many players [5]; this complexity is amplified in metazoans, which can possess thousands of different cell types that will impart varied effects on organismal aging. Because lineage-specific transcription factors will likely dictate the genes that ΔNp53:WTp53 regulates [8], we anticipate that the effects of ΔNp53:WTp53 expression will vary depending upon cell type. Such considerations represent interesting and important aspects for future work.

**Figure 1:**
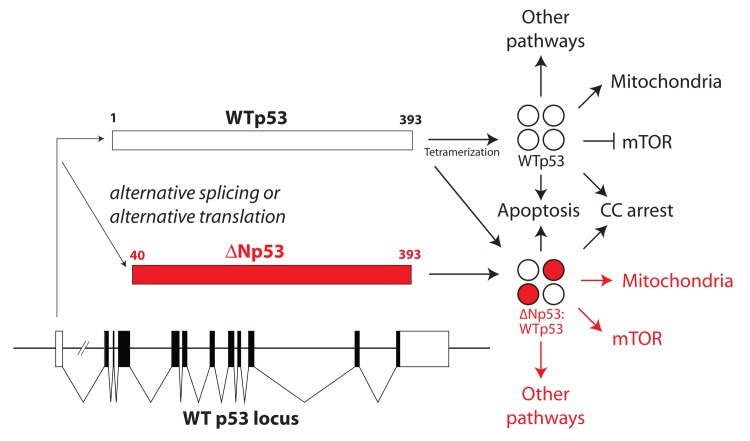
Overview of WTp53 and ΔNp53:WTp53 The ΔNp53 isoform is generated primarily through alternate translation but can also result from alternate splicing and even alternate transcription initiation. Generally speaking, mixed ΔNp53:WTp53 tetramers were similar to WTp53 in terms of their impact on H1299 (or Saos-2) cell growth, apoptosis, senescence, and gene expression (4). Notable exceptions included the mTOR pathway and mitochondrial function; additional pathways appear disrupted by ΔNp53:WTp53 expression and will be the subject of future experiments.
